# Supplementary material for: Detection of Antibiotic Resistance in Feline-Origin ESBL Escherichia coli from Different Areas of China and the Resistance Elimination of Garlic Oil to Cefquinome on ESBL E. coli
Source: Int J Mol Sci. 2023 Jun 1;24(11):9627. doi: 10.3390/ijms24119627 (PMC10253432; doi:10.3390/ijms24119627)
Supplement: Supplementary file 1 [file ijms-24-09627-s001.zip › ijms-2420602-supplementary.pdf]

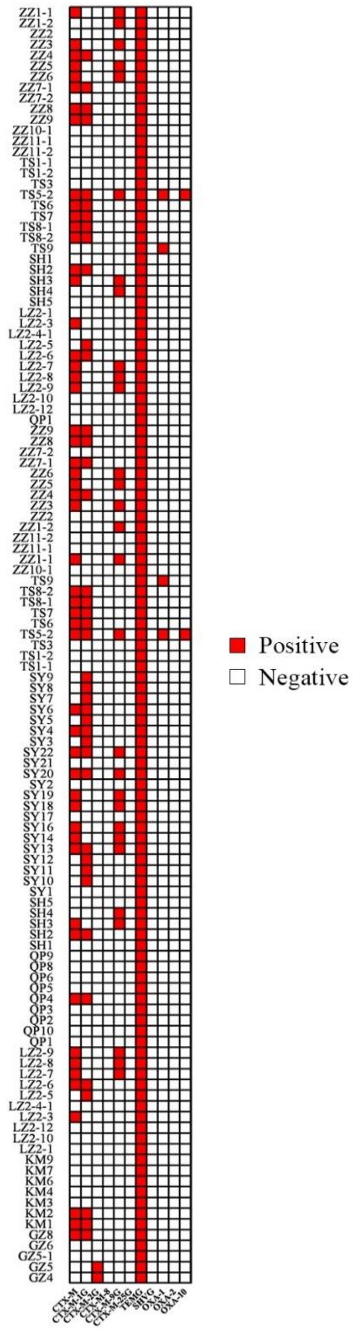

(A)

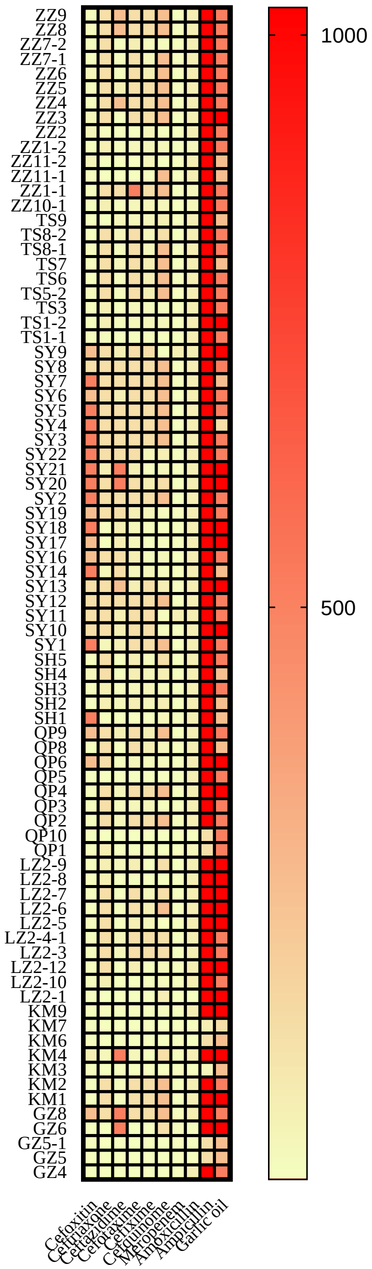

(B)

Figure S1. (A) Detection of ESBLs genes carried by 80 *E. coli* stains identified. **(B)** MICs of 80 identified *E. coli* strains for 9 antibiotics and garlic oil.
